# Establishment of cell-lines stably expressing recombinant Japanese eel follicle-stimulating hormone and luteinizing hormone using CHO-DG44 cells: fully induced ovarian development at different modes

**DOI:** 10.3389/fendo.2023.1201250

**Published:** 2023-08-25

**Authors:** Yukinori Kazeto, Risa Ito, Toshiomi Tanaka, Hiroshi Suzuki, Yuichi Ozaki, Koichi Okuzawa, Koichiro Gen

**Affiliations:** ^1^ Fisheries Technology Institute, Minamiizu Field Station, Japan Fisheries Research and Education Agency, Minamiizu, Shizuoka, Japan; ^2^ Fisheries Technology Institute, Tamaki Field Station, Japan Fisheries Research and Education Agency, Tamaki, Mie, Japan; ^3^ Hamanako Branch, Shizuoka Prefectural Research Institute of Fishery and Ocean, Hamamatsu, Shizuoka, Japan; ^4^ Fisheries Technology Institute, Shibushi Field Station, Japan Fisheries Research and Education Agency, Shibushi, Kagoshima, Japan; ^5^ Fisheries Technology Institute, Nagasaki Station, Japan Fisheries Research and Education Agency, Nagasaki, Japan

**Keywords:** recombinant follicle-stimulating hormone, recombinant luteinizing hormone, Japanese eel, ovarian development, fecundity

## Abstract

The gonadotropins (Gth), follicle-stimulating hormone (Fsh) and luteinizing hormone (Lh), play central roles in gametogenesis in vertebrates. However, available information on their differential actions in teleost, especially *in vivo*, is insufficient. In this study, we established stable CHO-DG44 cell lines expressing long-lasting recombinant Japanese eel Fsh and Lh with extra O-glycosylation sites (Fsh-hCTP and Lh-hCTP), which were produced in abundance. Immature female eels received weekly intraperitoneal injections of Gths. Fsh-hCTP induced the entire ovarian development by 8 weeks from the beginning of injection; thus, the ovaries of most fish were at the migratory nucleus stage while the same stage was observed in eels after 4 weeks in the Lh-hCTP-treated group. In contrast, all pretreated and saline-injected eels were in the pre-vitellogenic stage. Gonadosomatic indices in the Fsh-hCTP-treated group were significantly higher than those in the Lh-hCTP group at the migratory nucleus stage because of the significantly higher frequency of advanced ovarian follicles. Ovarian mRNA levels of genes related to E2 production (*cyp11a1*, *cyp17a1*, *cyp19a1*, *hsd3b*, *fshr*, and *lhr*) were measured using real-time quantitative reverse transcription-polymerase chain reaction (RT-PCR). All genes were induced by both Fsh-hCTP and Lh-hCTP, with a peak at either the mid- or late vitellogenic stages. Transcript abundance of *cyp19a1* and *fshr* in the Lh-hCTP group were significantly higher than those in the Fsh-hCTP group, whereas no difference in the expression of other genes was observed between the groups. Fluctuations in serum levels of sex steroid hormones (estradiol-17β, 11-ketotestosterone, and testosterone) in female eels were comparable in the Fsh-hCTP and Lh-hCTP groups, thus increasing toward the maturational phase. Furthermore, the fecundity of the eels induced to mature by Fsh-hCTP was significantly higher than that induced by Lh-hCTP. These findings indicate that Fsh and Lh can induce ovarian development in distinctively different modes in the Japanese eel.

## Highlights

- A mass production system of long-lasting recombinant Japanese eel Fsh and Lh, Fsh-hCTP and Lh-hCTP, was established.
- Both Fsh-hCTP and Lh-hCTP induced ovarian development from the pre-vitellogenic to migratory nucleus stage.
- Administration of Fsh-hCTP resulted in a higher gonadosomatic index than administration of Lh-hCTP at the migratory nucleus stage and overripe stage.
- Assisted reproduction using Fsh-hCTP resulted in higher fecundity than that using Lh-hCTP.

## 1 Introduction

In vertebrates, two gonadotropic hormones (Gths), follicle-stimulating hormone (Fsh) and luteinizing hormone (Lh), which are expressed in the pituitary gland, play important roles in reproductive biology (1). Fsh and Lh belong to the glycoprotein hormone family and form heterodimers in which the common α-subunit (Cga) and specific β-subunits (Fshb and Lhb) associate non-covalently. Fsh and Lh bind specifically to their respective receptors (Fshr and Lhr) expressed in gonadal somatic cells and regulate steroidogenesis and gametogenesis via the activation of transcription, translation, and post-translational modifications of target genes (2, 3).

Recent advancements in genome-editing technology have enabled gene targeting in fish, and Gth-knockout fish have been generated in two model fish species: zebrafish (4) and medaka (5). Analyses of the gonadal phenotypes of these knockout fish revealed species-specific differences in function, particularly in females. Fsh-deficient mutants were fertile in both sexes, whereas Lh was not essential for gonadal growth but was required for spawning in zebrafish. In contrast, Fsh and Lh are essential for follicle development and ovulation, respectively, and both Fsh- and Lh-deficient mutants are infertile in female medaka. In addition, Fsh and Lh were not required for spermatogenesis in the males of either species. However, the differences in the action of each Gth in non-model fish species, where genome-editing experiments are not performed frequently and/or take time to produce the experimental results, are not well understood (6). Furthermore, the difficulty in purifying sufficient amounts of Gths, especially Fsh, from fish pituitary glands and the lack of *in vivo* functional analysis is another major reason why the different function of each Gth in most species of fish is unknown. The production and use of recombinant Gths with biological activity *in vivo* are promising solutions to this problem.

To date, recombinant Gths from various fish species have been produced using a variety of expression systems (7–12). However, only recombinant Gths produced in mammalian cell lines have showed pronounced biological activity inducing gametogenesis *in vivo* (7, 13–15). Recently, we reported the production of Japanese eel single-chain recombinant Gths in a transient expression system, HEK293 cells, and their structure-*in vivo* activity relationship (9). The results demonstrated that the activity inducing spermatogenesis was much higher in recombinant Gths with an extra O-glycosylated site, a C-terminal peptide (hCTP) of β-subunit of human chorionic gonadotropin (hCG), than those for the others, due to the higher O-glycosylation and sialylation. However, *in vivo* experiments using the recombinant Gths cannot be repeated because of the limited amount of Gths produced by a transient expression system. Furthermore, it was reported that recombinant human coagulation factor VII expressed in HEK293 cells was less sialylated than that produced in CHO cells (16), suggesting that CHO cells rather than HEK293 cells are more suitable hosts for production of glycoproteins with high biological activity *in vivo*. Furthermore, CHO-DG44 cell line, a derivative of the CHO cell line, is dihydrofolate reductase (DHFR)-deficient, and this feature is known to enhance the expression of target recombinant proteins via amplification of the DHFR gene (17), ensuring the mass production of recombinant proteins.

In this study, a mass production system for recombinant eel Fsh and Lh was established using CHO-derived CHO-DG44 cells. We investigated whether there were specific differences in the action of both recombinant Gths with regard to fluctuations in body weight and gonadosomatic index (GSI), oocyte development, and activation of sex steroid and steroidogenesis-related genes, which are commonly used as indicators of maturation. In addition, the fecundity of artificially matured female eels with either Fsh or Lh was examined and compared.

## 2 Materials and methods

### 2.1 Establishment of CHO-DG44 cell-lines stably expressing Fsh-hCTP and Lh-hCTP

The construction of vectors encoding Fsh-hCTP and Lh-hCTP (pCAGGS-Fsh-hCTP and pCAGGS-Lh-hCTP) for transient protein expression was described in our previous report (9). The cDNA encoding the Fsh-hCTP was amplified by PCR using specific primers (P1: 5’-AGAGGATCCAACCCTTGCTAGCATGGATCTGGCTGTCACAG-3’ and P2: 5’-TAGGGATCCAACCCTTTCAATGGTGATGGTGATGATGACCGGT-3’) and a proofreading PrimeSTAR HS DNA polymerase (Takara, Tokyo, Japan), with pCAGGS-Fsh-hCTP as a template, and then subcloned into the pOptiVEC™-TOPO vector (Life Technologies, Carlsbad, CA, USA) for stable protein expression. The stable expression vector for Lh-hCTP was similarly constructed using P3, 5’-AGAGGATCCAACCCTTGCTAGCATGGCAGTCTACCCAGAATG-3’, and pCAGGS-Lh-hCTP, instead of P1 and pCAGGS-Fsh-hCTP, respectively. All the resultant vector constructs were bi-directionally sequenced using a 3130XL DNA sequencer (Applied Biosystems, Carlsbad, CA, USA) to confirm their validity.

A mammalian cell line stably expressing the target protein was established using the OptiCHO Express Kit (Life Technologies), conducted according to the manufacturer’s instructions, and the details were described in our previous report (18). Briefly, CHO-DG44 cells derived from dihydrofolate reductase-deficient Chinese hamster ovarian cells were cultured in CD DG44 medium (Life Technologies). Suspension cultures of CHO-DG44 cells were transfected with either the expression vector for Fsh-hCTP or Lh-hCTP, using the FreeStyle MAX Reagent (Life Technologies). After transfection, the transfected cells were passed in CD CHO Medium supplemented with L-glutamine to select stable transformants. Thereafter, the cells were maintained until their viability reached over 90%, and the resultant cell lines stably expressing Fsh-hCTP or Lh-hCTP were established at approximately 2-3 weeks after selection. The expression of Fsh-hCTP or Lh-hCTP was enhanced by genomic amplification by methotrexate selection (17).

### 2.2 Expression, purification and characterization of Fsh-hCTP and Lh-hCTP

The expression and purification of the target recombinant proteins were performed as described previously (9, 18). The culture conditions for stable cell lines expressing Fsh-hCTP and Lh-hCTP were optimized in CD OptiCHO Medium supplemented with GlutaMAX Supplement, using a CHO CD Efficient Feed Kit (Life Technologies). Cells of each stable line were cultured for 10-12 days under optimized conditions, and the media were harvested by centrifugation for subsequent purification of target proteins. After the concentration of the resultant media by ultrafiltration, the recombinant Gths were purified using immobilized metal affinity chromatography (IMAC) with Ni-NTA agarose (Qiagen, Valencia, CA, USA) and dialyzed with Dulbecco’s phosphate buffered saline (DPBS). The concentration of recombinant Gths was determined using the BCA protein assay kit (Pierce, Rockford, IL, USA).

Analysis of the resultant recombinant Gths was carried out as described previously (8) with some minor modifications. Fsh-hCTP and Lh-hCTP (5 μg/lane) were subjected to 12.5% SDS-PAGE under reducing conditions and stained using EzStain Aqua (ATTO, Tokyo, Japan). Western blot analysis with recombinant Gths (500 ng/lane) was also carried out after SDS-PAGE as described above. The proteins in the gels were electrophoretically transferred onto nitrocellulose membranes (Bio-Rad, Tokyo, Japan), and the membranes were incubated with rabbit anti-Cga, anti-Fshb, or anti-Lhb (8) of Japanese eel (1:2000) diluted in Can Get Signal Immunoreaction Enhancer Solution 1 (Toyobo, Tokyo, Japan) after blocking with 5% skim milk/TBS-T (20 mM Tris-HCl [pH 7.4], 150 mM NaCl, and 0.05% Tween 20), followed by incubation with goat anti-rabbit IgG conjugated with alkaline phosphatase (Funakoshi, Tokyo, Japan) diluted 1:2000 in Can Get Signal Solution 2. The coloring reaction was conducted using 4-nitro blue tetrazolium chloride and 5-bromo-4-chloro-3-indolyphosphate p-toluidine salt as chromogens. The concentrations of Fsh-hCTP and Lh-hCTP in the preparation after IMAC purification were measured by specific ELISA for eel Fsh and Lh, according to (19), and the purity was calculated based on the concentration of the recombinant Gth/concentration of the total protein x100.

### 2.3 Hormonal treatment of female Japanese eels *in vivo*

In this study, glass eels purchased from commercial suppliers were feminized with estradiol-17β (20) at the Hamanako Branch of the Shizuoka Prefectural Research Institute of Fishery. Eels do not consume food once they start to mature: therefore, fish were maintained without feeding from the start of hormone treatment.

#### 2.3.1 Experiment 1

Twenty-five immature female eels (BW=457.9 ± 22.1g) maintained in freshwater were used in this experiment. After acclimatization to seawater at a water temperature of 20°C, eels received weekly intraperitoneal injections of either Fsh-hCTP, Lh-hCTP or hCG at a dose of 500 µg/kg body weight (BW) or salmon pituitary extract (SPE) (20mg/kg BW) prepared according to (19), or DPBS as saline-injected control (n = 5 for each group). The body weight of each fish was measured at each time when fish were injected, and relative body weight (body weight/body weight at the 1st weekly injection × 100%) was calculated. Furthermore, the body weight was also measured 3 days after the last injection when the eel gained more than 15% at the time of injection. Eels were sampled when they gained 30% body weight 3 days after the last treatment. Fish that did not gain 30% body weight were sampled 3 days after the eighth injection. Fish were sacrificed after decapitation while under anesthesia (0.1% 2-phenoxyethanol) (Wako, Osaka, Japan), ovaries were removed and weighed, and the GSI (gonadal weight/body weight ×100) was determined. A portion of the ovary from each fish was collected and kept in DPBS to determine the developmental stage. Furthermore, some small fragments of the ovary were fixed using Bouin’s fixative.

#### 2.3.2 Experiment 2

Sixty-five immature female eels (BW=545.7 ± 21.9g) maintained in freshwater were used in this experiment. After acclimatization to seawater at 20°C, female eels (n = 5) served as pretreatment controls before hormonal treatment. Further eels received intraperitoneal injections of either Fsh-hCTP (n = 25) or Lh-hCTP (n = 25) at a dose of 500 µg/kg BW weekly or DPBS (n = 10) as saline-injected control, and blood samples were collected on a weekly basis. Gth-hCTP-treated eels were periodically sampled at 2, 4, 6, 8, and 10 weeks for Fsh-hCTP, and at 1, 2, 3, 4, and 5 weeks for Lh-hCTP (n = 5 at each time point) after decapitation under anesthesia. Eels from the control group (n = 5) were sacrificed at weeks 5 and 10. All sampling was performed 1 week after the last treatment.

The removed ovaries were weighed to calculate the GSI. A portion of the ovary from each fish was collected and kept in DPBS to determine the developmental stage. Furthermore, some small fragments of the ovary were fixed using Bouin’s fixative. Blood samples were collected for hormone measurement. Sera were obtained from the blood by centrifugation and stored at –30°C until analysis.

### 2.4 The classification of developmental stages of the ovary

Developmental stages of the ovary were classified as previously described (21) with some modifications. Ovarian fragments were loosened with fine forceps until the ovarian follicles could be clearly observed, and photographs were taken under a stereomicroscope. Then, using ImageJ (NIMH, Rasband, USA), 100 ovarian follicles were randomly selected from the photographs and their diameters were measured. The diameter of the largest group of follicles was calculated as the average diameter of the 20 largest follicles. Oocytes completed water absorption and became transparent, and oil droplets were clearly observed when the ovarian follicles grew to a diameter of 750-800 μm. For these follicles, the oil droplet diameter was measured, and the mean oil droplet diameter was calculated as an index for stage classification (22). Histological analysis of the ovary was conducted if the average diameter of the largest group of follicles was less than 400 μm. Ovarian tissues fixed with Bouin’s fixative were dehydrated using an ethanol series, embedded in paraffin, sectioned, and stained with hematoxylin and eosin. Ovaries were classified into the previtellogenic stage (PV) if no yolk globules were observed in the oocyte and the early vitellogenic stage (EV) if yolk globules were observed. The diameters of the largest group of follicles at the mid-vitellogenic stage (MV) were 400–600 μm, whereas those larger than 600 µm and prior to transparency were designated as the late vitellogenic stage (LV). Ovaries in which the transparency of the oocytes had been completed were classified into the migratory nucleus stage (MN), and overripe (OR) if the average oil droplet diameter in the oocytes was less than 190 µm and the average oil droplet diameter was greater than 190 µm, respectively.

The care and use of experimental animals was conducted in accordance with the guidelines of the Japan Fisheries Research and Education Agency and the guidelines set by the Japanese Ministry of Environment regarding standards for the care and use of laboratory animals, including the minimization of pain.

### 2.5 Frequency distribution of follicle diameter and percentage of the major group of the most advanced follicles

Frequency distributions of ovarian follicles of various diameters from each eel were assessed using 100 ovarian follicle diameters, measured as described above. A batch of the most developed follicles in the frequency distribution of follicle diameters was designated as the most advanced group. Furthermore, follicles with the most frequent diameter of ± 50 µm were designated as the major follicle group of the most advanced follicles. The proportion of the major follicle group of the most advanced follicles among all follicles was then estimated. Thirty ovarian follicles from the major follicle group were flash frozen in liquid nitrogen and stored at –80°C until analysis of gene expression.

### 2.6 Fertilization experiments

Twelve immature male eels (BW=449.5 ± 20.3g) cultivated from glass eels at the Hamanako Branch of the Shizuoka Prefectural Fisheries Research Institute were used in this experiment. Maturation of male eels was induced by Lh-hCTP administration, and milt was collected from each male according to our previous report (13). After confirming sperm motility activity, equal amounts of semen from 5-6 individuals were mixed and cryopreserved according to (23). The cryopreserved semen was thawed prior to use, diluted with K30 artificial seminal plasma consisting of 134.3 mM NaCl, 30 mM KCl, 20 mM NaHCO_3_, 1.6 mM MgCl_2_, and 1.3 mM CaCl_2_ buffered at pH 8.1 with 20 mM TAPS-NaOH (24), and used for insemination.

Ten immature female eels received intraperitoneal injections of either Fsh-hCTP or Lh-hCTP (500 µg/kg BW), as described above (n = 5 for each group). Eels were maintained at 20°C until week 3 and thereafter at 15°C because it has been reported that female eels should be maintained at 15°C prior to ovulation induction in order to facilitate the timing (25). When the fish had gained 15% body weight a day after the last treatment, a 1-2 mm hole was made in the abdomen using a surgical scalpel, from which the ovarian follicles were aspirated with a soft plastic cannula and observed under a stereomicroscope. Eels with ovarian follicles at MN were primed with Lh-hCTP (500 µg/kg BW), and the water temperature was increased to 20°C. One day later, fish were intraperitoneally injected with 17α-hydroxyprogesterone (17α-OHP: Sigma, St. Louis, MO, USA), a precursor of eel maturation-inducing steroid (17α,20β-dihydroxy-4-pregnen-3-one), at a dose of 2 mg/kg BW to induce final oocyte maturation and ovulation. Fourteen hours after the 17α-OHP injection, the first check of ovulation conducted by squeezing the abdomen of the female eels. From females that had ovulated, eggs were gently stripped and immediately inseminated with sperm samples as described above. For fish for which ovulation was not confirmed, this process was continued at an interval of 1-2 hours until 18 hours after 17α-OHP injection. The eggs obtained from each eel were weighed and the number of eggs per 1 g was counted. The number of eggs per 500 g BW of fish before hormone treatment was calculated as relative fecundity.

The detailed procedures for artificial insemination have been described previously (23). The rates of fertilization, hatching, and survival of larvae at 6 days after hatching (stage at the first feeding) were evaluated using a microplate method (26).

### 2.7 Real-time quantitative RT-PCR

Total RNA from the 30 ovarian follicles of the major follicle group of the most advanced follicles was extracted by the guanidium thiocyanate-phenol-chloroform extraction method using ISOGEN (NIPPON GENE, Toyama, Japan). cDNA was synthesized from the total RNA (500 ng) after priming with random hexamers, using ReverTra Ace qPCR RT Master Mix with gDNA Remover (TOYOBO, Osaka, Japan) according to the manufacturer’s instructions. The transcript abundance of steroidogenic enzyme genes (*cyp11a1*, *hsd3b*, *cypc17*, *cyp19*) and Gth receptor genes (*fshr* and *lhr*) in ovarian follicles was determined by real-time quantitative RT-PCR using Gene Ace Probe qPCR Mix α Low ROX (NIPPON GENE) or GeneAce SYBR qPCR Mix Low Rox (NIPPON GENE) on an Applied Biosystems 7500 Real-Time PCR System, according to the manufacturer’s instructions. The design of specific primers and probes for each gene was previously described (27–29). The real-time quantitative RT-PCR conditions and validity followed the ABI PRISM Sequence User Guide and previously published reports (27–29). Transcript abundance of genes was expressed as the number of copies of transcripts per ovarian follicle for steroidogenic enzymes and Gth receptors.

### 2.8 Time-resolved fluoroimmunoassay

Serum concentrations of sex hormones (E2, 11-KT and T) were measured by time-resolved fluorescence immunoassay (TR-FIA) according to the methods described elsewhere (30, 31).

### 2.9 Statistical analysis

All numerical data are presented as mean ± standard error of the mean. One-way analysis of variance followed by Fisher protected least significant difference *post hoc* test was used to determine significant differences among respective means between the time points and developmental stages in each experimental group. Further, Student’s t-test was carried out to detect significant differences among respective means at each time point and in the same developmental stage between experimental groups. Differences were considered statistically significant at *p* < 0.05.

## 3 Results

### 3.1 Purification and characterization of recombinant Gths

**Figure 1:**
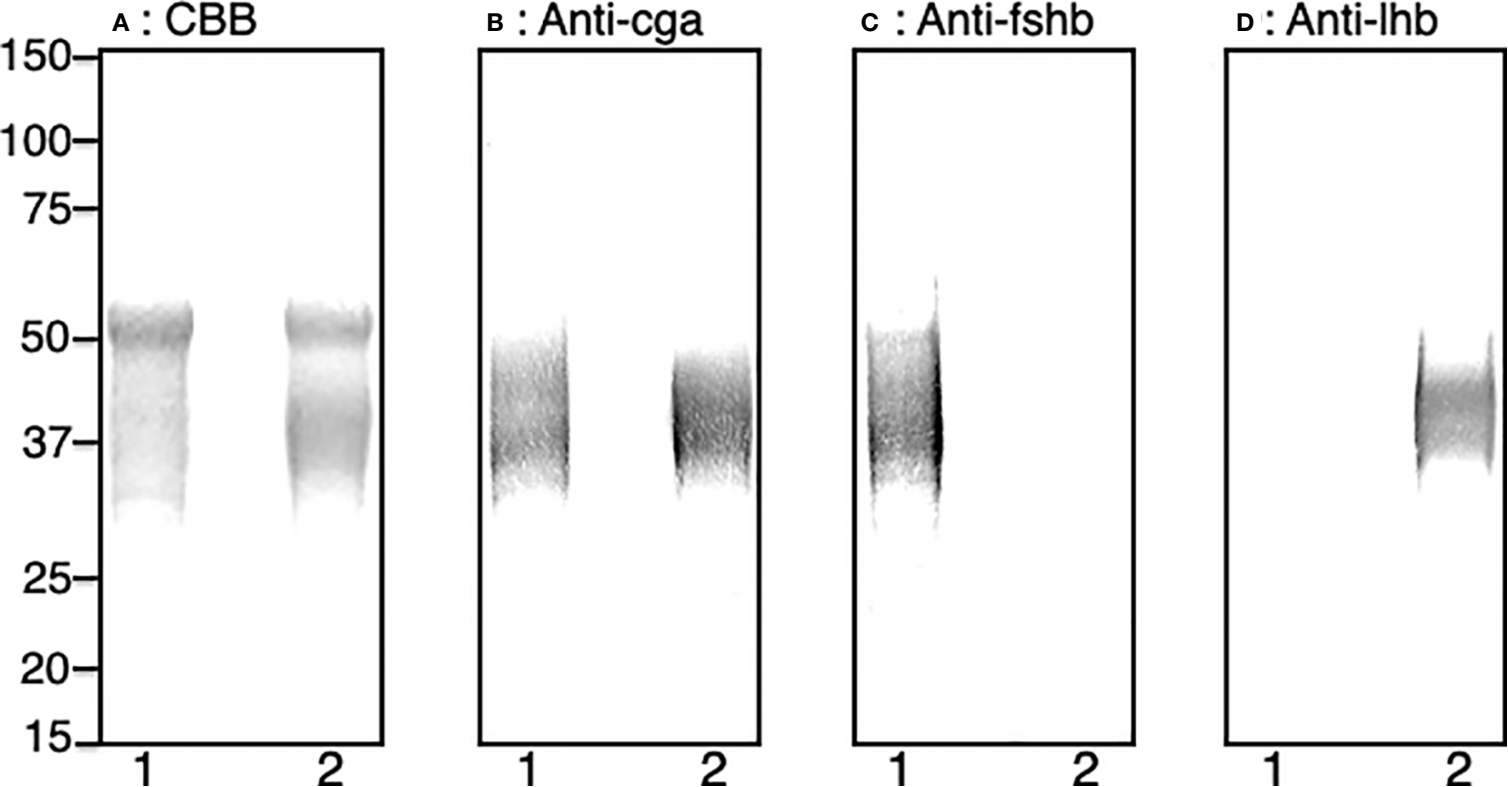
SDS-PAGE and western blot analyses of the purified recombinant Gths produced by CHO-DG44 cells. **(A)** Total protein staining, **(B)** western blot with anti-Cga, **(C)** western blot with anti-Fshb, **(D)** western blot with anti-Lhb. Lane 1: Fsh-hCTP; lane 2: Lh-hCTP.

Fsh-hCTP and Lh-hCTP, stably expressed in CHO-DG44 cells, were purified and analyzed by SDS-PAGE under reducing conditions. A blurry band was detected at 30-49 kDa for Fsh-hCTP and 31-46 kDa for Lh-hCTP by total protein staining (**Figure 1A**). An intense band with a molecular mass of 50-51 kDa was also observed in the preparations of Fsh-hCTP and Lh-hCTP. Western blot analyses using anti-Cag detected immunoreactive signals against the blurry band of both Fsh-hCTP and Lh-hCTP (**Figure 1B**), whereas analysis using each anti-β-subunit only detected the corresponding recombinant Gths (**Figures 1C, D**). The intense band with a molecular mass of 50-51 kDa did not provide any immunoreactive signal in either the Fsh-hCTP or Lh-hCTP preparations. The purities of Fsh-hCTP and Lh-hCTP were determined as approximately 50-55%. The yields of these recombinant Gths ranged between 5 and 10 mg/L-medium.

### 3.2 Effects of Gth-hCTP on body weight and ovarian development in Japanese eels *in vivo*

#### 3.2.1 Experiment 1

**Figure 2:**
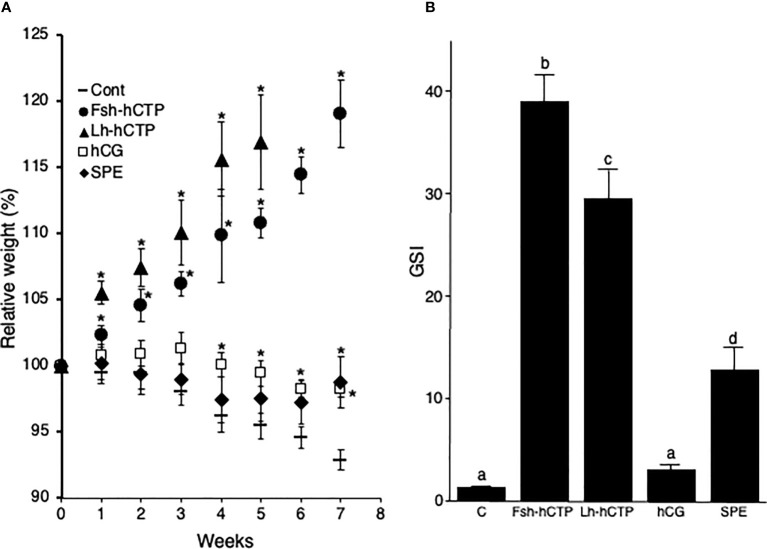
Fluctuations in relative weight of female eels during treatment **(A)**, and GSI **(B)** in each experimental group at the end of the treatment, in Experiment 1. Values are expressed as mean ± standard error. Relative weight is calculated using the weight at the start of the experiment as 100%. (*) indicates significant difference (*p* < 0.05) between the levels before the treatment and at each time point among the same treatment group. Different lettering above bars indicates significant differences (*p* < 0.05) among respective means.

Changes in relative weight of female eels at each time of injection is shown in **Figure 2A**. While the weight of the control group gradually decreased over time, that of Gth-hCTP group showed a marked increase (**Figure 2A**). The Fsh-hCTP- and Lh-hCTP-treated groups showed a statistically significant increase in weight compared to the saline-injected group from the first week of treatment throughout the experimental period. The Lh-hCTP group showed a faster weight increase, the relative weight reached 115-120% at time of injection at 4-5 weeks and all eels had gained over 30% body weight 3 days after the last injection, and sampled. In contrast, most of the eels (80%) in the Fsh-hCTP group reached a 30% weight increase 3 days after the last injection at 7 weeks. In contrast, the hCG- and SPE-treated groups maintained weight or lost weight gradually but to a slower degree than the control group. The hCG and SPE groups showed significantly higher body weights than the control group from week 4 onward and at week 7, respectively. There was no significant difference in the GSI between the control and hCG groups. However, in the other treatments, GSI was significantly higher than that in the control group, and was highest in Fsh-hCTP, followed by Lh-hCTP and SPE (**Figure 2B**).

All fish in the control group were in the PV whereas vitellogenesis was induced in all fish in the other experimental groups. In the Fsh-hCTP and Lh-hCTP groups, 80% of the individuals were in the MN and 20% in the LV. In contrast, all fish in the hCG group were in the EV whereas 20%, 20%, and 60% of fish in the SPE group were in the EV, MV and LV, respectively.

#### 3.2.2 Experiment 2

**Figure 3:**
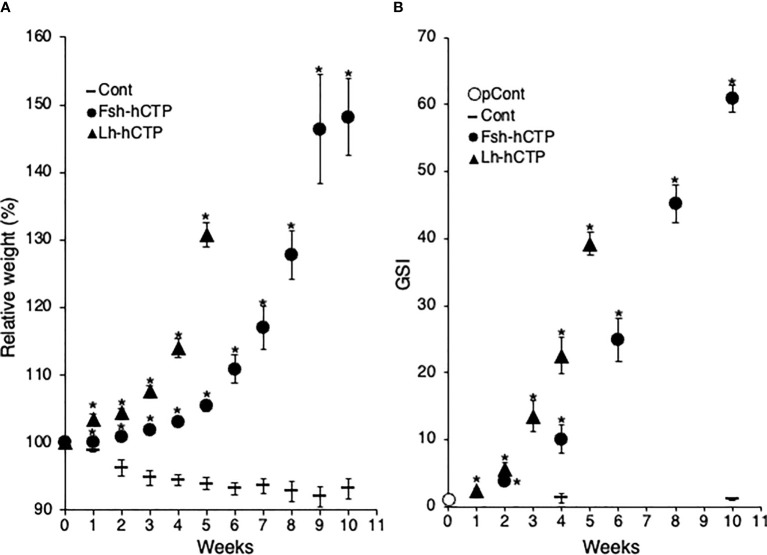
Fluctuations in relative weight **(A)** and GSI **(B)** of female eels during treatment in Experiment 2. Values are expressed as mean ± standard error. (*) indicates significant difference (*p* < 0.05) between the levels before the treatment and at each time point among the same treatment group.

The weight of fish in the saline-injected control group decreased throughout the experimental period, as in Experiment 1, whereas the Fsh-hCTP- and Lh-hCTP groups showed an increase in weight, and significant differences were detected compared to those in the control group throughout the experimental period. As in Experiment 1, the relative weight of the eels in the Lh-hCTP group reached over 130% at week 5 (**Figure 3A**). In contrast, the Fsh-hCTP group showed gradual weight gain, reaching 127.8% relative weight at week 8 and then increasing to 148.2% by week 10 (**Figure 3A**). The change in GSI over time during the experiment is shown in **Figure 3B**. There was no significant difference in GSI between the pretreatment control and saline-injected control (week 4 and week 10) groups, whereas the GSI in the Gth-hCTP-treated groups was significantly higher than that in the pre-treatment control group at all time points.

**Figure 4:**
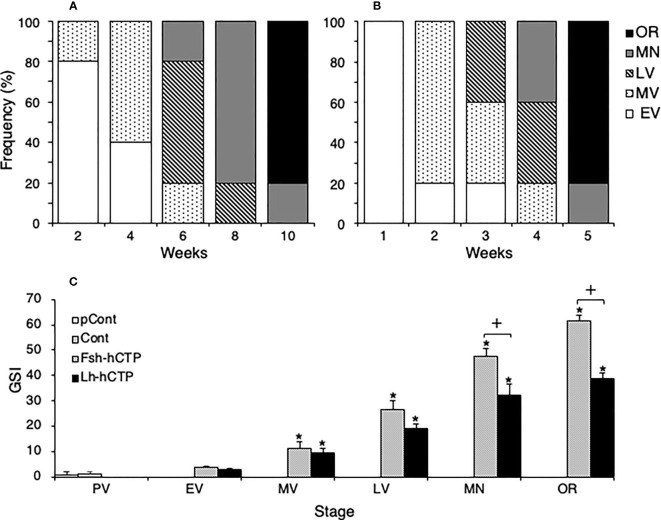
Changes in the appearance frequency of ovaries at each developmental stage during Fsh-hCTP **(A)** or Lh-hCTP **(B)** treatment, and comparison of GSI at the same ovarian developmental stage in each experimental group **(C)**. (*) indicates significant difference (*p* < 0.05) between the levels before the treatment and at each time point among the same treatment group. (+) indicates significant difference (*p* < 0.05) among respective means in the same developmental stage between experimental groups.

All fish in the pretreatment control and saline-injected control groups were in the PV. Changes in the developmental stages of ovaries treated with Fsh-hCTP or Lh-hCTP are shown in **Figures 4A, B**, respectively. In the Fsh-hCTP group, the developmental stages of the ovaries progressed over time: dominant stage was EV at week 2, MV at week 4, LV at week 6, MN at week 8, and OR at week 10, (**Figure 4A**). In contrast, the ovaries in the Lh-hCTP group developed faster than those in the Fsh-hCTP group, reaching each developmental stage in approximately half of the treatment period (**Figure 4B**). The GSI of both the pretreatment and saline-injected control groups was approximately 1, whereas in both the Fsh-hCTP and Lh-hCTP groups, the GSI increased rapidly with the progression of ovarian developmental stages, reaching its highest value at OR (**Figure 4C**). A comparison of GSI at each developmental stage in the Fsh-hCTP and Lh-hCTP groups showed similar values up to MV, but a trend toward higher GSI in the Fsh-hCTP group was observed in the LV. The GSI of the Fsh-hCTP group was significantly higher than that of the Lh-hCTP group in the MN and OR.

### 3.3 Frequency distribution of follicle diameter and percentage of the major group of the most advanced follicles

**Figure 5:**
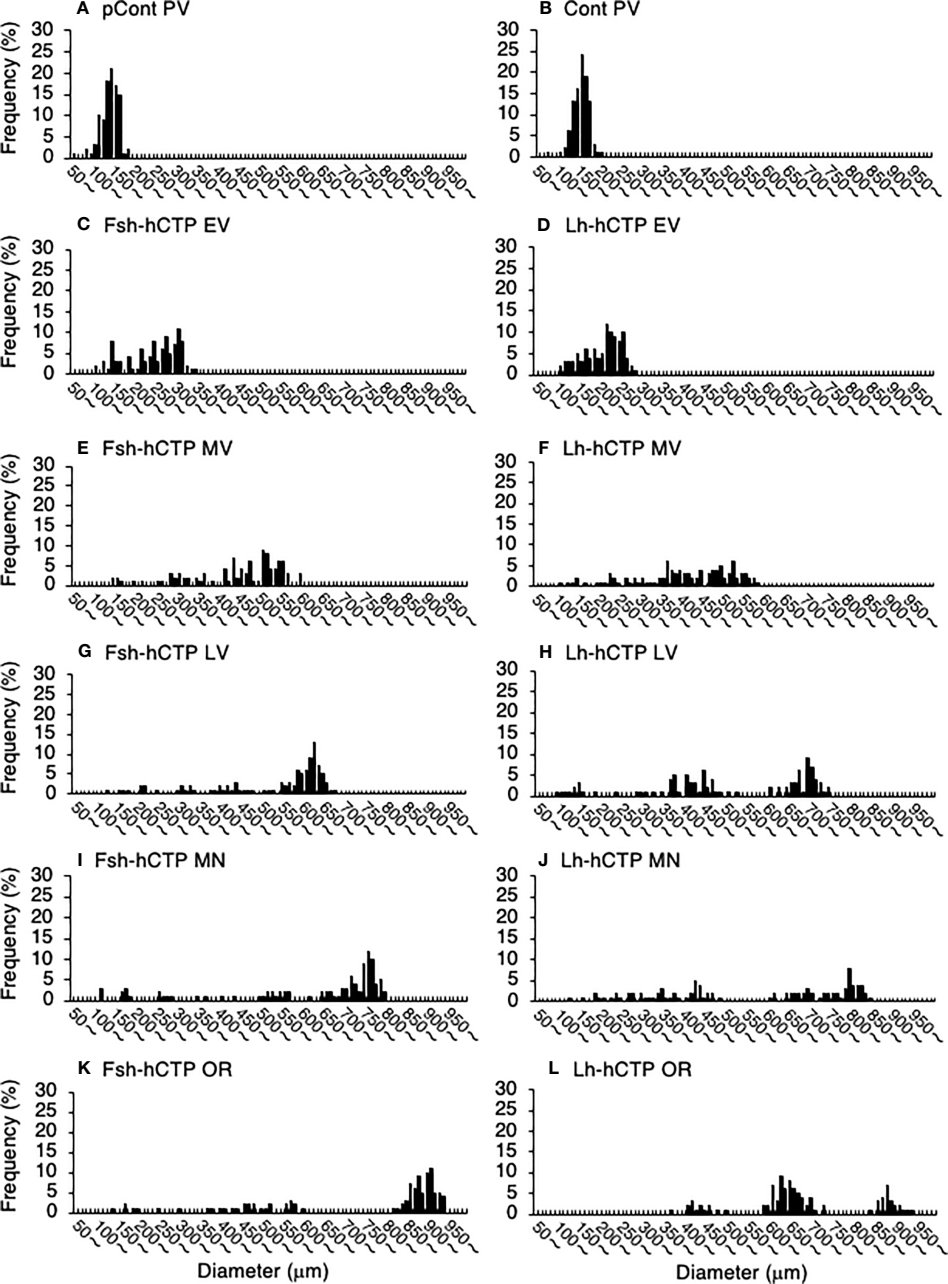
Representative frequency distributions of ovarian follicles of various diameters in ovaries at different developmental stages in each experimental group. **(A)** PV in pretreatment control group (pCont); **(B)** PV in saline-control group (Cont); **(C)** EV in Fsh-hCTP group; **(D)** EV in Lh-hCTP group; **(E)** MV in Fsh-hCTP group; **(F)** MV in Lh-hCTP group; **(G)** LV in Fsh-hCTP group; **(H)** LV in Lh-hCTP group; **(I)** MN in Fsh-hCTP group; **(J)** MN in Lh-hCTP group; **(K)** OR in Fsh-hCTP group; and **(L)** OR in Lh-hCTP group.

**Figure 6:**
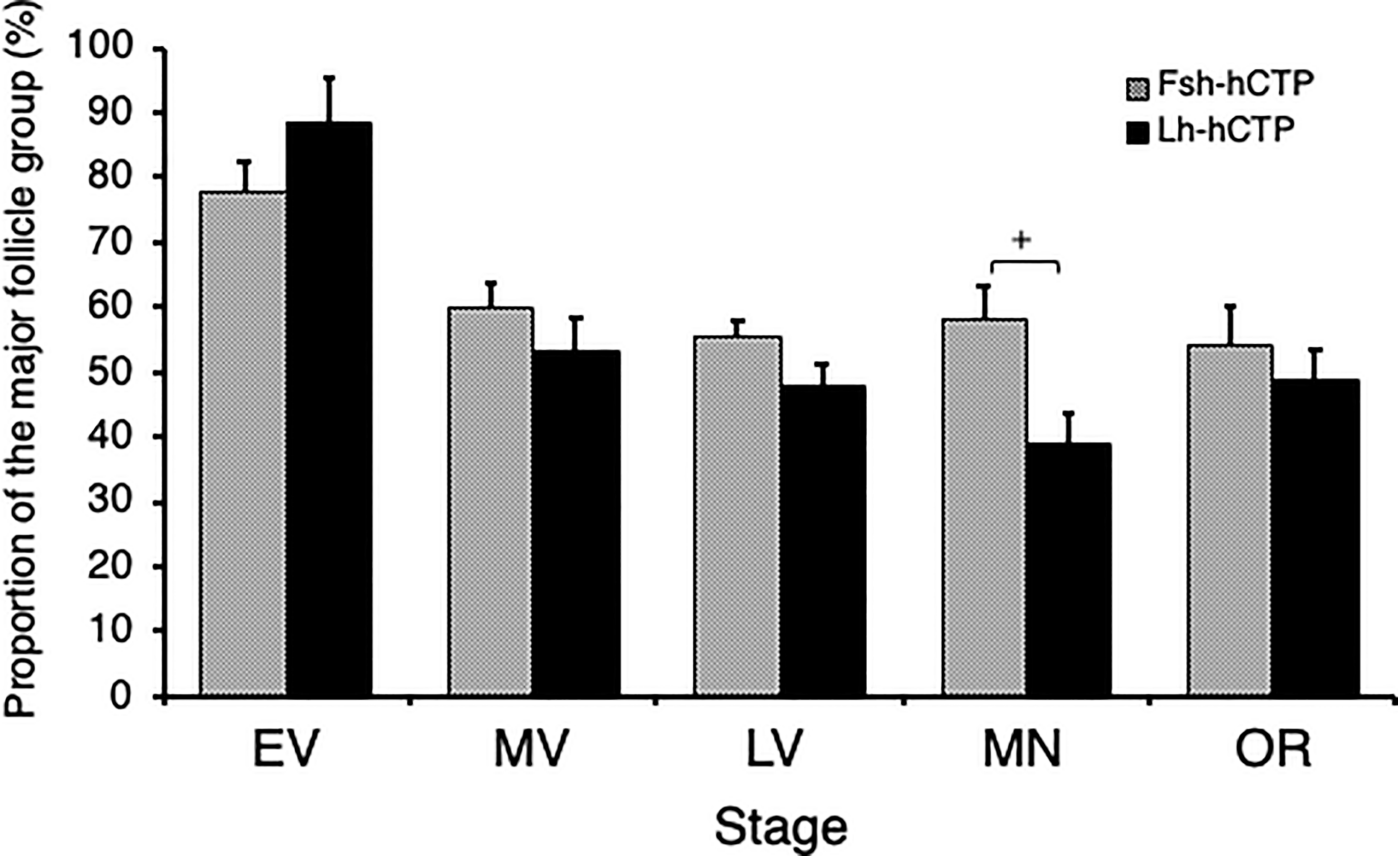
Proportion of the major ovarian follicles of the most advanced follicle group in ovaries at each developmental stage. (+) indicates significant difference (*p* < 0.05) among respective means in the same developmental stage between experimental groups.

Representative frequency distributions of ovarian follicles of various diameters in the ovaries at various developmental stages in each experimental group are shown in **Figure 5**. One distinct batch of ovarian follicles was observed in the ovaries at PV from fish in the pre-treatment control group and the saline-injected control group (**Figures 5A, B**). The batch of the most advanced follicles became less distinct as the ovaries developed into the EV and MV after Fsh-hCTP or Lh-hCTP treatment (**Figures 5C–F**). Subsequently, a cluster of the most advanced follicles was clearly observed in the ovaries from the LV to the OR (**Figures 5G–L**), and this trend was particularly pronounced in the FSH group. The proportion of the major follicle group was significantly higher (approximately 1.5-fold) in the Fsh-hCTP group than in the Lh-hCTP group when the ovaries were at the MN (**Figure 6**).

### 3.4 Fecundity and parameters of quality of eggs in eels reproductively assisted with Gth-hCTP

Ovulation was induced in all fish used and the resultant eggs were subjected to artificial fertilization experiments. Injection times of Fsh-hCTP and Lh-hCTP required for fertilization experiment were 9.0 ± 1.3 and 8.2 ± 0.6, respectively. Duration from 17α-OHP injection to egg retrieval was 14.4 ± 0.3 in Fsh-hCTP group and 15.2 ± 0.3 in Lh-hCTP group. Relative fecundity was significantly higher in female eels artificially matured using Fsh-hCTP than in Lh-hCTP-treated eels (**Table 1**). Similarly, the fertilization rate of the resultant eggs in Fsh-hCTP group was significantly greater than that in Lh-hCTP group. The hatching and survival rates of larvae at 6 days post-hatching in the Fsh-hCTP group also tended to be higher in Fsh-hCTP group than those in Lh-hCTP group although the differences were not significant.

**Table 1:** Values related to egg collection, fecundity and egg quality.

	Fsh-hCTP (n=5)	Lh-hCTP (n=5)
Injection times to reach MN stage	9.0 ± 1.3	8.2 ± 0.6
Duration from 17α-OHP injection to egg retrieval (hr)	14.4 ± 0.3	15.2 ± 0.3
Relative fecundity^1^	39.8 ± 2.7	26.0 ± 2.7**
Fertilization rate (%)	77.7 ± 11.9	41.0 ± 9.8*
Hatching rate (%)	36.6 ± 13.3	3.5 ± 2.6
Survival rate of larvae at 6 days post hatching (%)	34.4 ± 13.6	3.4 ± 2.6

^1^: egg numbers/10^4^/500g-BW.

**: indicates statistical significance between Fsh-hCTP and Lh-hCTP (Student’s t-test, p<0.01).

*: indicates statistical significance between Fsh-hCTP and Lh-hCTP (Student’s t-test, p<0.05).

### 3.5 Expression of genes related to steroidogenesis in ovarian follicle

**Figure 7:**
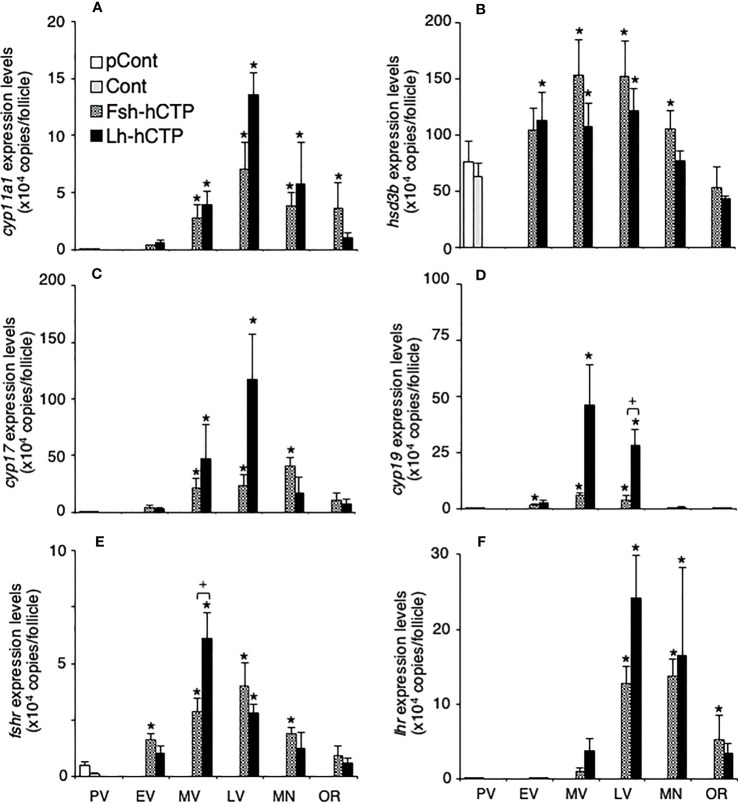
Transcript abundance of genes of steroidogenic enzymes and Gthrs during ovarian development induced by Gths. Transcript abundance was determined by real-time quantitative RT-PCR and expressed as copy numbers per ovarian follicle. **(A)** *cyp11a1*; **(B)** *hsd3b*; **(C)** *cyp17*; **(D)** *cyp19*; **(E)** *fshr*; **(F)** *lhr*. (*) indicates significant difference (*p* < 0.05) between the levels at PV (before the treatment) and at each developmental stage among the same treatment group. (+) indicates significant difference (*p* < 0.05) among respective means in the same developmental stage between experimental groups.

Transcript abundance of *hsd3b* was relatively high in the ovarian follicles at PV, whereas the other genes showed very low expression levels (**Figures 7A–F**). After the start of Gth-hCTP treatment, the expression levels of all genes increased significantly, reaching their highest levels in the MV or LV, and then decreased. The gene expression levels of *cyp19* (**Figure 7D**) and *fshr* (**Figure 7E**) were significantly higher in the Lh-hCTP group than in the Fsh-hCTP group at the LV and MV, respectively, when comparing the expression levels of each gene at the same developmental stage between the Fsh-hCTP- and Lh-hCTP-treated groups.

### 3.6 Changes in serum levels of steroid hormones during ovarian development

**Figure 8:**
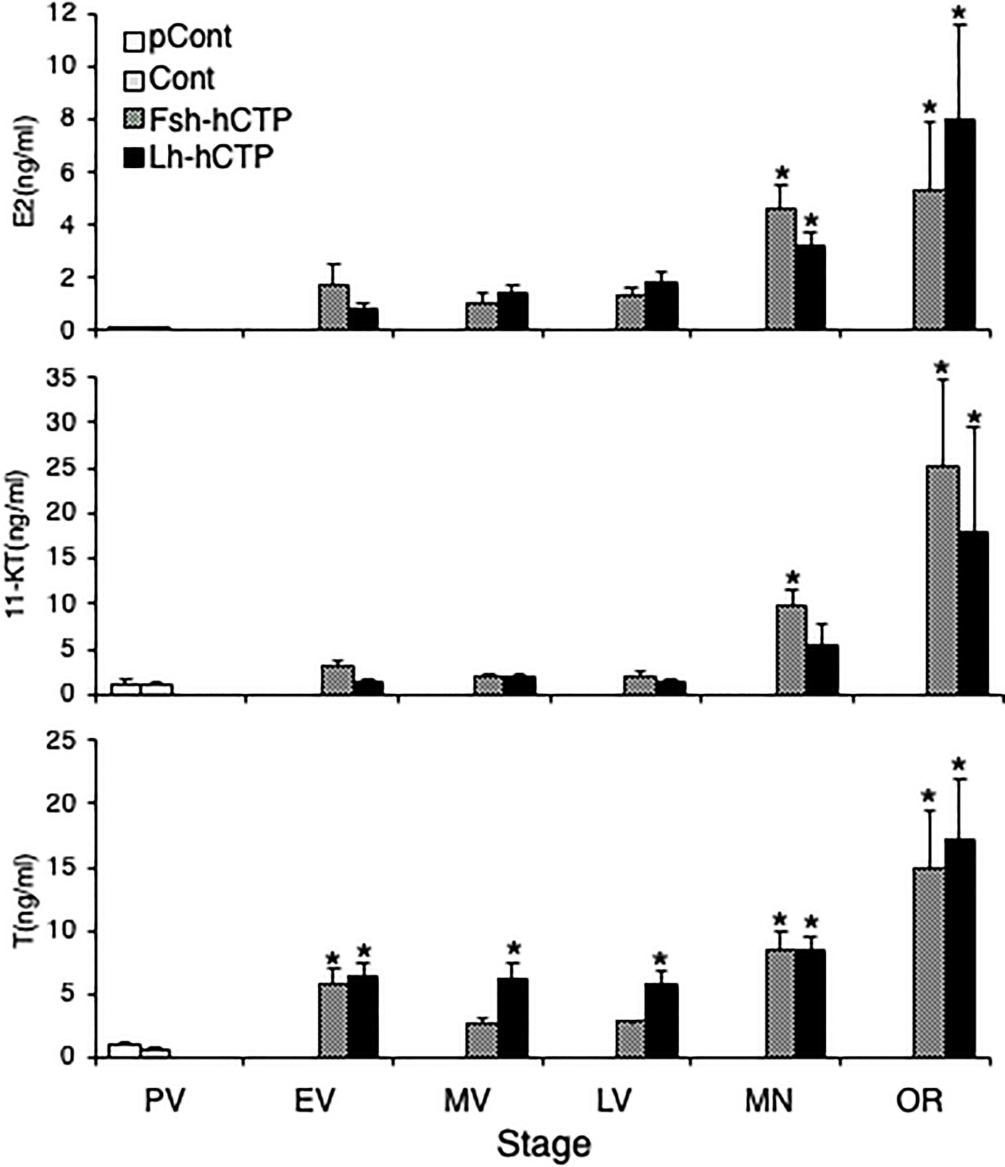
Serum levels of steroid hormones during ovarian development induced by Gths. (*) indicates significant difference (*p* < 0.05) between the levels at PV (before the treatment) and at each developmental stage among the same treatment group.

Serum E2 levels were low in PV in the pretreatment and saline-injected control groups, and thereafter tended to increase in EV to LV after Gth-hCTP administration, with statistically significant increases in MN and OR. The serum levels of 11-KT and T also showed changes similar to those of E2 (**Figure 8**). There was no statistical difference in serum steroid levels between the Fsh-hCTP and Lh-hCTP groups at the same developmental stage.

## 4 Discussion

In this study, stable cell lines expressing recombinant Japanese eel Gths with an extra O-glycosylation site (Fsh-hCTP and Lh-hCTP) were successfully established, and Gth-hCTPs were produced on a large-scale. Furthermore, their differential actions on female reproductive biology, for example, development of ovaries and oocytes, expression of genes related to reproduction, and fecundity were examined *in vivo*.

Both Fsh-hCTP and Lh-hCTP purified using IMAC contained a band (50-51 kDa) that was not immunoreactive against antibodies to all subunits of Japanese eel Gths, indicating the resultant Gth-hCTPs were crude preparations. This contaminant was also purified from the conditioned media of wild-type CHO-DG44 cells (data not shown), strongly suggesting that it was not a Gth-associated protein. Furthermore, its properties (molecular weight, isoelectric point, etc.) did not appear to be significantly distinguishable from those of eel Gth-hCTPs, and contaminants could not be easily removed from crude Gth-hCTPs by gel filtration, ion exchange chromatography, or Con-A Sepharose chromatography. Therefore, we decided to use crude Gth-hCTPs in this study. Both Fsh-hCTP and Lh-hCTP were detected as blurred bands by both total protein staining and specific western blotting, as reported previously in the case of the corresponding Gth-hCTPs produced by another expression system using HEK293 cells (19). The molecular masses of Fsh-hCTP (30-49 kDa) and Lh-hCTP (31-46 kDa) were also comparable to those in the previous report, and the yields (5-10 mg/L-medium) were high enough. Taken together, these results suggested that Gth-hCTPs were produced correctly and abundantly, as expected.

The biological activities of Fsh-hCTP and Lh-hCTP were examined in immature female Japanese eels *in vivo*. First, Fsh-hCTP and Lh-hCTP, as well as SPE and hCG which have been used to induce maturation in female and male eels (32), were administered to immature female eels to examine and compare their effects on indicators of maturation in female eels, the body weight and GSI (Experiment 1). Although there were differences between the groups, the saline-treated control, hCG-treated, and SPE-treated groups showed a gradual decrease in body weight, suggesting that maturation did not progress or progressed poorly. In contrast, body weight increased rapidly in the Gth-hCTP groups, particularly in the Lh-hCTP group, suggesting that newly produced recombinant Gth-hCTPs strongly induced ovarian development and possessed greater bioactivity than gonadotropic reagents used in Japanese eels. In fact, the GSIs in the Fsh-hCTP and Lh-hCTP groups were 25- to 30-fold higher than those in control group. SPE induced ovarian development and increased GSI, as shown in many previous reports (19–21, 27, 32) but the values were only approximately 10-fold higher than those in the control group. In contrast, the hCG group showed a slight increase in GSI without statistical significance. The developmental stages of the ovary in each group coincided with the corresponding GSI; the stages in the Gth-hCTP groups were most advanced, followed by the SPE, hCG and control groups. Since all individuals in the hCG group were at the EV while all in the control group were at the PV, it was considered that hCG can induce vitellogenesis, but the effect is very limited. Fsh activates only Fshr, while Lh and SPE induce signal transduction through both Fshr and Lhr in Japanese eels (8). Furthermore, our previous report demonstrated that hCG-induced activation was restricted to Lhr, but not Fshr (28). Therefore, it was concluded that the activation of Fshr, but not Lhr was indispensable for efficient vitellogenic growth of oocytes in the Japanese eel, and Lh-hCTP appeared to mainly induce vitellogenesis via Fshr.

It has been proven that full maturity can be achieved by Fsh-hCTP and Lh-hCTP administration *in vivo*, and the oogenesis process induced by both was further examined in detail. In Experiment 2, more eels subjected to Gth-hCTP administration were periodically sampled and analyzed. Based on the results of Experiment 1, it was expected that ovarian development was induced faster in the Lh-hCTP group than in the Fsh-hCTP group. To confirm this hypothesis, in Experiment 2, the Lh-hCTP group was given a shorter treatment period than the Fsh-hCTP group and a shorter sampling interval. As in Experiment 1, body weight decreased gradually in the control group but increased rapidly in the Gth-hCTP group. Especially in the Lh-hCTP group, the increase in body weight was more rapid than that in the Fsh-hCTP group. In contrast, the Fsh-hCTP group showed a slower increase in body weight, but the maximum value was higher than that of the Lh-hCTP group. The GSI fluctuations were also in good agreement with body weight fluctuations. Ovarian developmental stages predominantly observed at each time point in the Fsh-hCTP group were observed in the Lh-hCTP group at approximately half the duration of treatment. Thus, all phenomena indicate that Lh-hCTP has a greater ability to induce ovarian development than Fsh-hCTP in Japanese eels. As reported in other fish species (33, 34), it has been shown that Fsh activates only Fshr in Japanese eels, whereas Lh binds to both Fshr and Lhr to express its hormonal activity (28). The high ovarian growth-promoting effect of Lh in the Japanese eel could thus be attributed to its ability to activate both receptors. However, this hypothesis contradicts the extremely low ability of hCG, an agonist that activates only Lhr, to induce vitellogenesis. The role of Lhr signaling in vitellogenic growth remains to be elucidated.

Interestingly, the GSI in the Fsh-hCTP group tended to be higher at the LV than that in the Lh-hCTP group and was significantly higher thereafter, even though the ovarian developmental stage was the same. A detailed analysis of the frequency distribution of ovarian follicles of various diameters showed that the proportion of major ovarian follicles (follicles with the most frequently appearing diameter ± 50 µm) in the most advanced follicle group tended to be higher in the Fsh-hCTP-treated group at LV and was significantly higher (approximately 1.5-fold) at MN. This may be the reason for the higher GSI of mature ovaries in the Fsh-hCTP group than that in the LH group. To further confirm this hypothesis, egg retrieval experiments were conducted using females matured by either Fsh-hCTP or Lh-hCTP. This is the first trial of egg retrieval from female eels matured by recombinant Gth, therefore we referred to the already established method of ovulation induction in female eels matured with SPE (32). In this method, eels at MN are further primed with SPE, and finally, the maturation-inducing steroid or the precursor (17α-OHP) is administered. In addition, since it is well known that Lh plays an important role in the final oocyte maturation and ovulation, Lh-hCTP was used instead of SPE for priming in this report. This method could induce ovulation in all eels. The relative fecundity was significantly and approximately 1.5-fold higher in the Fsh-hCTP group than in the Lh-hCTP group, which is in good agreement with the increase in the proportion of the major advanced ovarian follicles in the Fsh-hCTP group. In addition to the present study, induction of the entire process of oogenesis from previtellogenic individuals by administration of Gths was reported in flathead grey mullet (*Mugil cephalus*) (15). However, this report was aimed at seedling production of mullet and does not provide any information on the different actions of Fsh and Lh in oogenesis, as our report does. For deeper understanding of the mechanism of action of Gths on fish oogenesis, it is essential to establish a system for mass production of highly active Gths *in vivo* also in other fish species, and to analyze the different effects of Fsh and Lh on oogenesis *in vivo*. Regarding the quality of the resultant eggs, fertilization rate was significantly higher in the Fsh-hCTP group than in the Lh-hCTP group, and other parameters also tended to be greater in the Fsh-hCTP group. In the Fsh-hCTP group, Fsh-hCTP and Lh-hCTP were administered in sequence to induce oocyte growth and final maturation/ovulation, respectively. The order of hormone administration in the Fsh-hCTP group was physiologically relevant, since it is well known in teleost that Fsh and Lh play important roles in oocyte growth and maturation, which likely made the quality of eggs better in the Fsh-hCTP group than that in the Lh-hCTP group. Considering the higher fecundity, artificial induction of ovarian development using Fsh-hCTP appears to be more suitable than Lh-hCTP for seedling production in Japanese eels. The quality of the eggs obtained in this study was comparable to that reported previously when eels were subjected to assisted reproduction with heterologous gonadotropic reagents such as SPE (22, 25). To obtain eggs of better quality, further optimization of assisted reproduction with recombinant Gths remains an important issue.

Sex steroid hormones are major intermediates in Gth-induced oogenesis. In order to elucidate some of the mechanisms of the different actions of Fsh and Lh in oogenesis, the expression of steroid production-related genes and fluctuations in serum levels of steroid hormones were examined. Gene expression of steroidogenic enzymes and Gthrs in the most advanced ovarian follicles was upregulated at the MV or LV and then decreased. Similar gene expression analyses have been reported in ovarian fragments induced to mature by SPE treatment using real-time quantitative RT-PCR or northern blotting (21, 27, 28, 35–37). With the exception of *hsd3b*, upregulation of gene expression as vitellogenesis was induced by administration of gonadotropic reagents, which has been shown in previous reports and is consistent with the present results (21, 27, 28, 35–37). However, most genes exhibited different expression patterns after MV. Transcript abundance of *cyp11a1*, *cyp17*,and *cyp19* genes increased from MV to MN (21, 35, 36). Furthermore gene expression of *hsd3b* did not change during ovarian development (37). The reason for this difference could be due to the difference in gonadotropic reagents administered, the method to determine gene expression, and/or differences in the experimental samples, that is, ovarian fragment vs. separated ovarian follicles. In contrast, fluctuations in gene expression of *fshr* and *lhr* during ovarian development in this study was comparable to that reported elsewhere (28); thus, transcript abundance of *fshr* peaked at the LV, while *lhr* gene expression was rapidly induced at the LV and remained at a high level thereafter. There were no marked differences in the expression of steroidogenesis-related genes between the Fsh-hCTP and Lh-hCTP groups; significant differences were detected only at certain stages in *cyp19* and *fshr*. In fact, the changes in serum levels of sex steroid hormones, as the endpoint of the changes in the gene expression, were comparable in both groups. Therefore, the above-mentioned difference in fecundity between the Fsh-hCTP and Lh-hCTP groups is unlikely to be due to differences in steroid production capacity or steroid hormone levels.

Since this is the first report on *in vivo* changes in gene expression of steroidogenic enzymes with the development of ovaries induced by homologous Gths, it cannot be compared with cases in other species of teleosts. However, the changes in the gene expression of steroidogenic enzymes in the ovary during the natural reproductive cycle have been demonstrated in other teleost species. In rainbow trout, the expression levels of *cyp11a1*, *cyp17*, and *cyp19* increased in the latter half of vitellogenesis and decreased rapidly after vitellogenic completion, whereas the expression level of *hsd3b* did not change significantly during vitellogenesis and decreased slightly after completion (38). In channel catfish (*Ictalurus punctatus)*, the expression levels of *cyp11a1*, *cyp17*, and *hsd3b* increased rapidly in the LV, whereas the transcript abundance of *cyp19* increased with the onset of vitellogenesis and remained high during vitellogenesis. Thereafter, the expression levels of all four genes decreased after the end of vitellogenesis. The gene expression patterns in the rainbow trout and channel catfish appeared to be comparable to those in Japanese eels, as shown in this study. Furthermore, the changes in the expression of *gthr* genes in the eel ovary are mostly consistent with those reported in zebrafish (39) and channel catfish (40, 41) during naturally occurring reproductive cycles. Thus, the changes in steroidogenic enzyme genes during ovarian development, whether induced by the administration of Gths or occurring under natural conditions, are similar, which suggests that assisted reproduction using recombinant Gths induces ovarian development/steroidogenesis just as it occurs naturally.

In summary, a mass production system of recombinant Japanese eel Fsh-hCTP and Lh-hCTP was successfully established using the CHO-DG44 expression system, which enabled us *in vivo* analyses to clarify the differential actions of Fsh and Lh in gonadal development and steroidogenesis. Lh-hCTP had a higher ability to induce ovarian development than Fsh-hCTP, whereas Fsh-hCTP led to higher fecundity, which did not appear to be due to differences in steroidogenesis. The regulatory mechanism of fecundity in teleosts is unknown, and further studies using eels as a model should be conducted to gain a deeper understanding of reproductive regulation by Gths.

## Data availability statement

The raw data supporting the conclusions of this article will be made available by the authors, without undue reservation.

## Ethics statement

Ethical review and approval was not required for the fish study because this study was conducted in accordance with the guidelines of the Japan Fisheries Research and Education Agency and the guidelines set by the Japanese Ministry of Environment regarding standards for the care and use of laboratory animals, including the minimization of pain. In this case, the committee did not require us the approval.

## Author contributions

YK and KG designed the study. YK and YO produced and analyzed recombinant hormones. YK, and RI, KO, TT and HS conducted *in vivo* experiment. RI performed steroid measurement and qRT-PCR analysis. YK wrote the manuscript. All authors contributed to the article and approved the submitted version.
